# Concurrent resection and orthognathic surgery for the management of mandibular condyle osteoma: a case report and literature review

**DOI:** 10.3389/froh.2026.1777630

**Published:** 2026-07-30

**Authors:** Dhafer M. Alsuwied, Mody Alhomied, Albraa B. Alolayan

**Affiliations:** 1 Department of Dentistry, King Faisal Specialist Hospital and Research Center, Jeddah, Saudi Arabia; 2 College of Medicine, Alfaisal University, Riyadh, Saudi Arabia; 3 Department of Oral and Maxillofacial Surgery, Faculty of Dentistry, University of Toronto, Toronto, ON, Canada; 4 Department of Oral and Maxillofacial Diagnostic Sciences, Dental College and Hospital, Taibah University, Madinah, Saudi Arabia

**Keywords:** case report, facial asymmetry, mandibular condyle, orthognathic surgery, osteoma

## Abstract

Facial asymmetry is a common sequela of condylar osteoma and has a significant impact on patient's quality of life. In some cases, sole resection of the tumor and surrounding structures is insufficient to restore maxillofacial harmony, and incorporating additional surgical procedures, such as orthognathic surgery, might provide a more comprehensive solution. This paper reports the case of a 36-year-old woman who presented with pain on biting on the right side, accompanied by swelling over the right periauricular region. Clinical examination revealed facial asymmetry with a chin shift to the left, limited mouth opening, a right posterior open bite, and canting of the occlusal plane. The simultaneous performance of condylar osteoma resection and orthognathic surgery, supported by virtual surgical planning, provided a comprehensive surgical approach to address the patient's condition while reducing overall treatment and recovery times by avoiding the need to perform these two procedures separately.

## Introduction

Osteomas are considered rare benign tumors, with an estimated frequency of 0.01%–0.04%. The peak incidence of osteomas lies between the ages of 30 and 50 years, with no sex predilection (1, 2). These tumors typically develop in bone formed by intramembranous ossification and are most frequently found in the craniofacial skeleton, particularly in the paranasal sinuses, where their prevalence reaches 3.69% (3, 4). Osteomas can be classified into three types based on their tissue of origin: extra-skeletal (from muscle), peripheral (from periosteum), and central (from endosteum), with the peripheral type being the most commonly encountered (5).

When osteomas occur in the jaws, they most often present as peripheral osteomas and have a higher predilection for the mandible than the maxilla (2). Osteomas arising in the mandibular condyle can pose a serious dilemma. Their critical location at the articulation between the mandible and the temporal bone can significantly disrupt the architecture of the temporomandibular joint (TMJ) (6). These tumors may result not only in unaesthetic facial deformities but also in substantial functional impairment (7).

Despite their clinical implications, the rarity of condylar osteomas (COs) has limited the development of new evidence-based treatment strategies and reduced clinicians’ confidence in managing complex cases. In addition, the literature lacks data on the simultaneous management of CO and facial asymmetry (FA) using a combined surgical approach that integrates osteoma resection with orthognathic surgery (OS). The aim of this study is to report a rare case of mandibular CO with FA treated by simultaneous osteoma resection and orthognathic surgery supported by virtual surgical planning (VSP) and to review the existing literature on the clinical presentation and management of this condition.

## Case description

A 36-year-old Saudi woman presented to King Faisal Specialist Hospital and Research Center in Jeddah, Saudi Arabia, with a 4-year history of pain on biting on the right side and a swelling over the right preauricular region. She denied any medical history and previous trauma to the head and neck region. Her past surgical history was significant for a diaphragmatic hernia repair performed 2 years earlier, which resulted in a hypertrophic scar.

**Figure 1:**
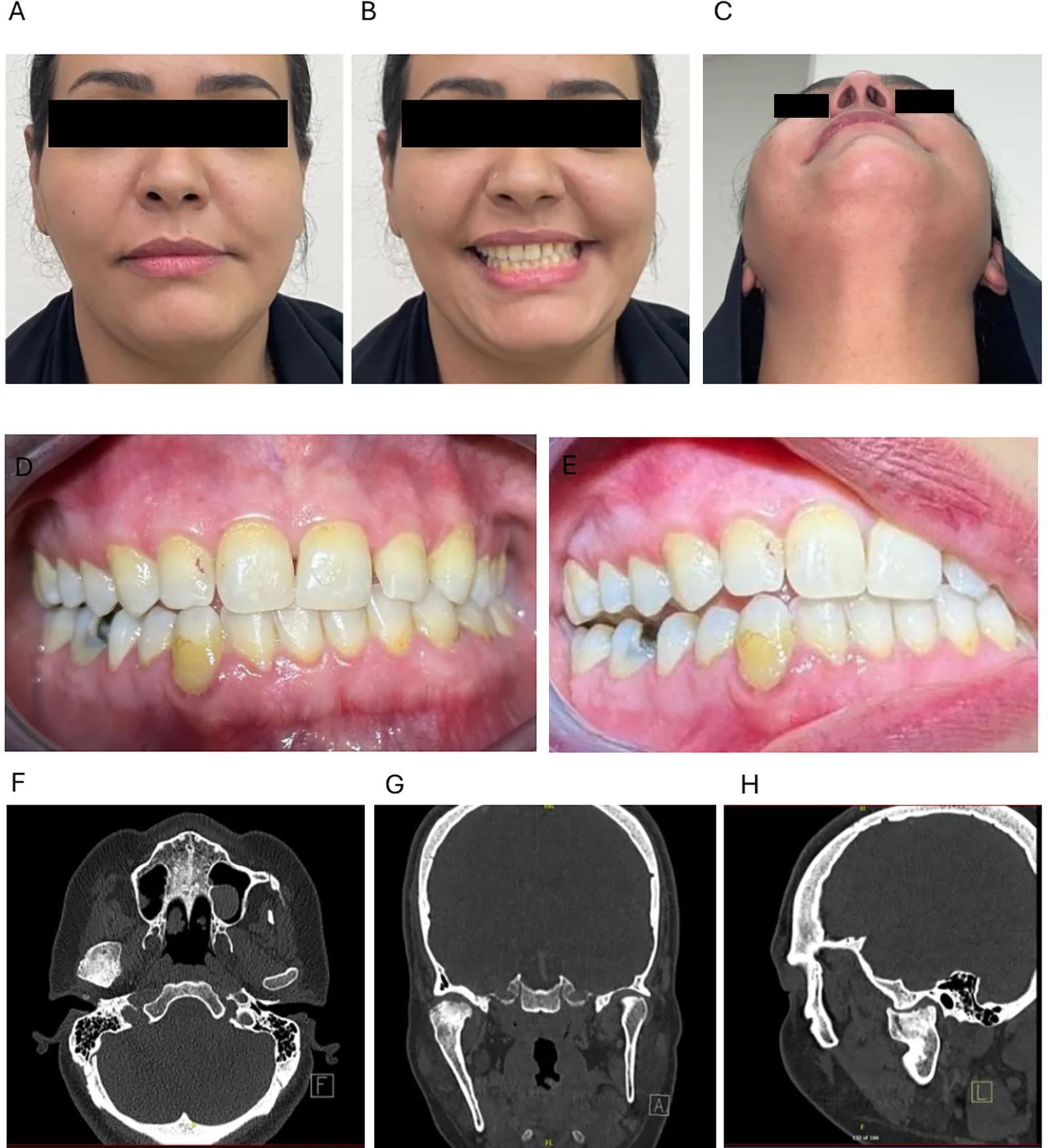
Photographs of the patient depicting FA with chin deviation to the left: **(A)** frontal view at rest, **(B)** frontal view with a smile, **(C)** submental (worm's eye) view, **(D)** canting of maxillary and mandibular occlusal planes downward on the right side with a mandibular midline shift to the left, **(E)** open bite in the right posterior segment, **(F)** CT axial plane, and medial extension of the CO evident on the **(G)** coronal plane and **(H)** sagittal plane.

On general examination, the patient was obese and appeared fairly nourished. Extraoral examination of the head and neck region revealed FA, with the chin deviated to the left side, which became more prominent on smiling (Figures 1A–C). A firm, bony swelling was palpable in the right preauricular region, with normal overlying skin. No regional lymph nodes were clinically palpable. Intraoral examination showed a maximum interincisal opening of two fingers, a class III subdivision right molar relation, a unilateral right posterior open bite, and downward canting of the maxillary and mandibular occlusal planes on the right side, which was more pronounced at the mandibular arch. A 2–3 mm mandibular-to-maxillary dental midline shift to the left was noted (Figures 1D,E).

Preoperative imaging included an orthopantomogram, lateral and posteroanterior cephalograms, and computed tomography (CT) scans of the head and neck. The orthopantomogram revealed an irregular mass of radiodensity and trabeculation similar to surrounding bone, extending from the anterosuperior aspect of the right condylar head to the anterior slope of the articular eminence. The lateral cephalogram demonstrated a Class I skeletal pattern, while the posteroanterior cephalogram confirmed FA. CT imaging revealed enlargement and sclerosis of the right condylar process measuring 2.3 cm × 1.9 cm × 1.1 cm in maximum anteroposterior, transverse, and craniocaudal dimensions, respectively, with loss of architecture and a narrow zone of transition. Degenerative changes were also noted in TMJ articular surfaces (Figures 1F–H). A few prominent cervical lymph nodes were noted bilaterally in group IIB, most likely reactive in nature, as the architecture was preserved.

These findings posed a diagnostic challenge due to overlap between certain clinical and radiographic features and other pathologic and neoplastic entities. The differential diagnosis list included CO, condylar osteoarthritis, condylar hyperplasia, and, least likely, osteosarcoma. A biopsy was not attempted because the history, clinical and radiographic findings, and the benign features noted were sufficient to establish a preliminary diagnosis of CO.

**Figure 2:**
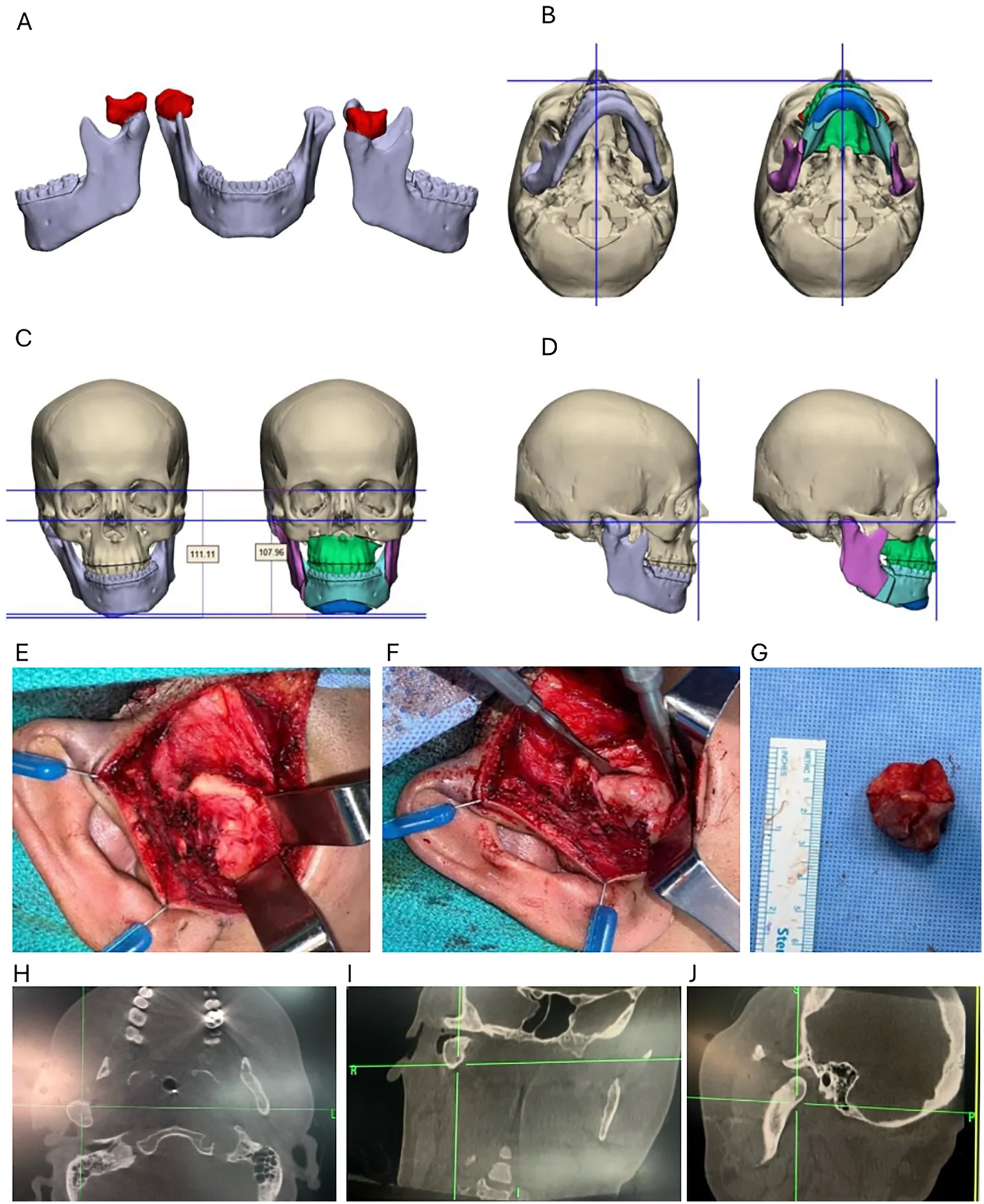
VSP comparing initial patient presentation with the final plan of tumor resection, Le Fort I osteotomy, bilateral sagittal split osteotomies, and genioplasty. (**A**) CO and its resection margins delineated in red: (**B**) axial view, (**C**) frontal view, and (**D**) lateral view; (**E**) exposure of the tumor; (**F**)condyle following the tumor resection; (**G**) extirpated specimen; and intraoperative postresection CT images acquired by the O-Arm to confirm complete tumor resection: (**H**) axial view, (**I**) coronal view, and (**J**) sagittal view.

VSP was performed to coordinate the resection and orthognathic procedures: Le Fort I, bilateral sagittal split, and genioplasty. Three-dimensional CT data were exported in Digital Imaging and Communications in Medicine format. Intraoral occlusion scanning was performed using Trios3 (3Shape, Copenhagen, Denmark), and the data were exported in Standard Tessellation Language format. The datasets were imported into Materialise software (Materialise NV, Leuven, Belgium) to plan the osteotomies and final occlusion (Figures 2A–D). A conservative marginal resection was planned due to the benign and well-defined characteristics of the tumor and the absence of reported recurrence following resection of COs; the extent of resection was determined by the tumor margins, which were identified based on the obvious alteration and deviation from the normal condylar contour and anatomy. Patient-specific titanium cutting guides and their corresponding custom titanium plates were designed and fabricated for the osteotomies.

### Surgical technique

Under general anesthesia with nasoendotracheal intubation, the patient was prepared and draped in the standard fashion. The right condyle was approached through an Al-Kayat preauricular incision, which was carried through the skin and subcutaneous tissue to the temporalis fascia. Blunt superior dissection was carried out toward the zygomatic arch until the superficial layer of the temporalis fascia was incised. The periosteum of the zygomatic bone was then incised, followed by blunt inferior dissection superficial to the TMJ capsule. Anterior dissection was continued until the articular eminence was reached. The TMJ capsule was incised, exposing the articular disc, tumor, and condyle. The tumor was then conservatively resected using an oscillating saw, and the sharp bony edges were smoothed (Figures 2E–G). Intraoperative O-Arm imaging was used to evaluate the resection and confirm complete removal of the osteoma (Figures 2H–J).

Second, the maxilla was approached. Local anesthesia consisting of 10 cc of 2% lidocaine 1:100,000 epinephrine was injected in the maxillary vestibule, followed by a conventional Le Fort I osteotomy technique using a custom cutting guide. Correction of the maxillary cant involved a 2.5-mm descent on the left side and a 1.6-mm ascent on the right side. A 1.7-mm advancement and 3.3 mm posterior impaction were completed, along with clockwise yaw correction. Fixation was achieved using 15 screws (1.5 mm) and two 0.8-mm custom titanium plates. Next, intermaxillary fixation (IMF) was achieved using elastics and six IMF screws.

Third, attention was directed to the mandible. Similarly, local anesthesia was injected in the mandibular vestibule, followed by a conventional bilateral sagittal split osteotomy performed using custom cutting guides. The mandible was advanced by 1.3 mm and rotated 1.3 mm clockwise. Fixation was achieved with eight screws and two 1-mm custom titanium plates. Occlusion was verified as stable following release of the IMF.

Finally, using a custom cutting guide, the chin was advanced by 0.44 mm and rotated 3.12 mm clockwise. Fixation utilized four screws and a 0.8-mm custom titanium plate.

Closure was performed as follows: the right preauricular incision was closed in layers using 3–0 vicryl for deep layers and 4–0 prolene for skin closure, an alar cinch suture was placed using 3–0 polydioxanone (PDS), and the intraoral maxillary vestibule, V–Y closure and mandibular vestibular incision were closed using 3–0 vicryl. Triamcinolone (2 cc) was injected into the preauricular incision site after closure. Heavy elastics were applied over the IMF screws. A chin pressure dressing together with Steri-Strips over the preauricular wound was applied.

**Figure 3:**
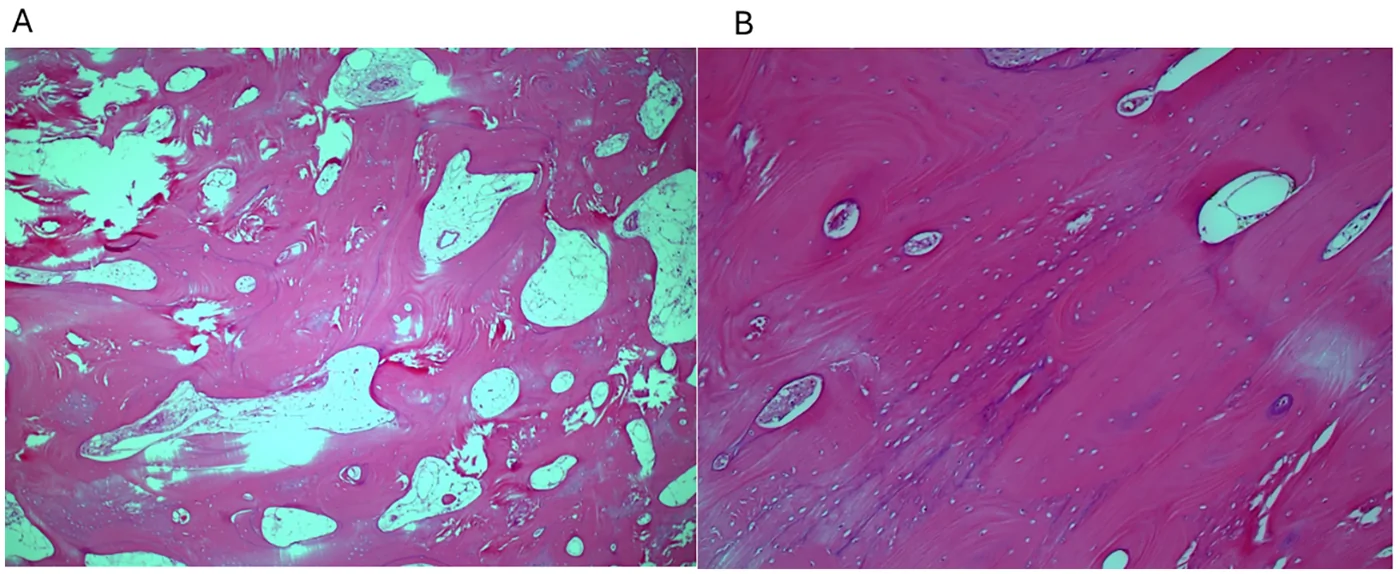
Histologic sections of the extirpated specimen. **(A)** Mature lamellar bone with a compact and spongy pattern; **(B)** osteocytes in lacuna without atypia or significant osteoblastic activities [H&E staining; **(A)** ×40 magnification; **(B)** ×100 magnification].

Intraoperative specimens of four fragments of reddish white hard bony tissue measuring 4.0 cm × 3.4 cm × 0.9 cm in aggregate were submitted for histopathologic examination. Microscopic examination revealed mature lamellar bone with a well-demarcated fibrotic rim, consistent with an osteoma. No osteoblastic proliferation, atypical cellular components, or infiltrative growth was identified (Figures 3A,B). The absence of cytological atypia and radiographic features indicative of a destructive growth pattern makes osteosarcoma an unlikely diagnosis (8). Condylar osteoarthritis, a degenerative condition, typically presents with joint pain, mandibular dysfunction, and radiographic signs of degeneration, subchondral cysts, and osteophyte formation, none of which were observed in this patient (9). Furthermore, the exophytic morphology and histologic lack of a cartilaginous cap argue against condylar hyperplasia, which usually demonstrates uniform enlargement of the condylar head and a cartilage cap covering the condylar surface (10). Taken together, the clinical, radiographic, and histologic findings established the final diagnosis of solitary peripheral osteoma of the right mandibular condyle.

### Postoperative course and follow-Up

Following surgery, the patient developed right-sided frontal branch of facial nerve weakness and lower lip and chin hypoesthesia. Improvement of the frontal nerve was first noted on day 4. The patient was discharged on postoperative day 6 in a stable condition.

**Figure 4:**
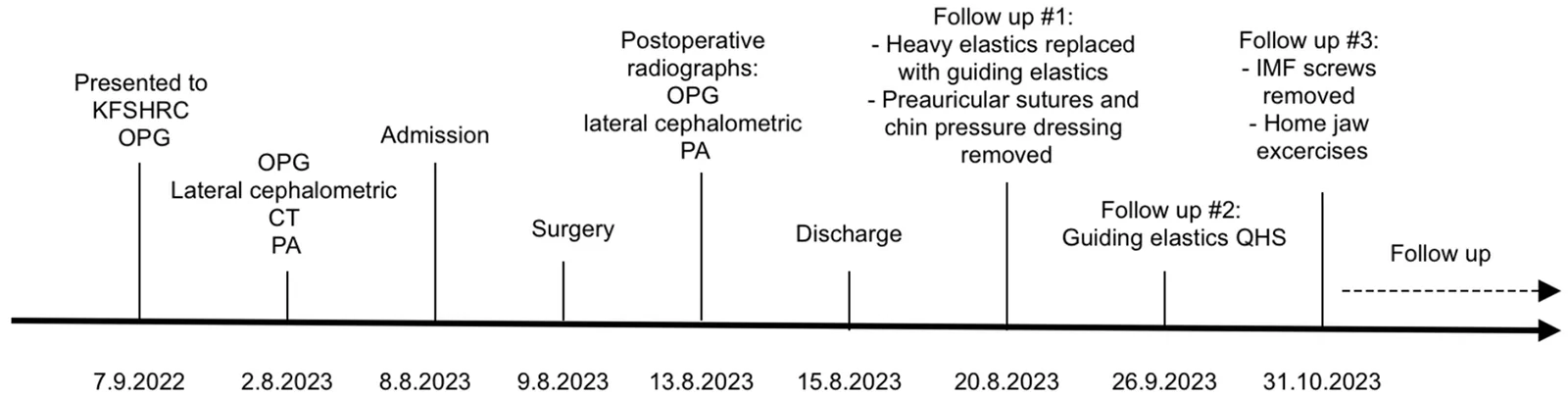
Timeline of the patient's diagnostic work-up, treatment planning, surgical intervention, and follow-up. KFSHRC, King Faisal Specialist Hospital and Research center; OPG, orthopantogram; CT, computed tomography; PA, posteroanterior radiograph; QHS, nightly; IMF, intermaxillary fixation.

**Figure 5:**
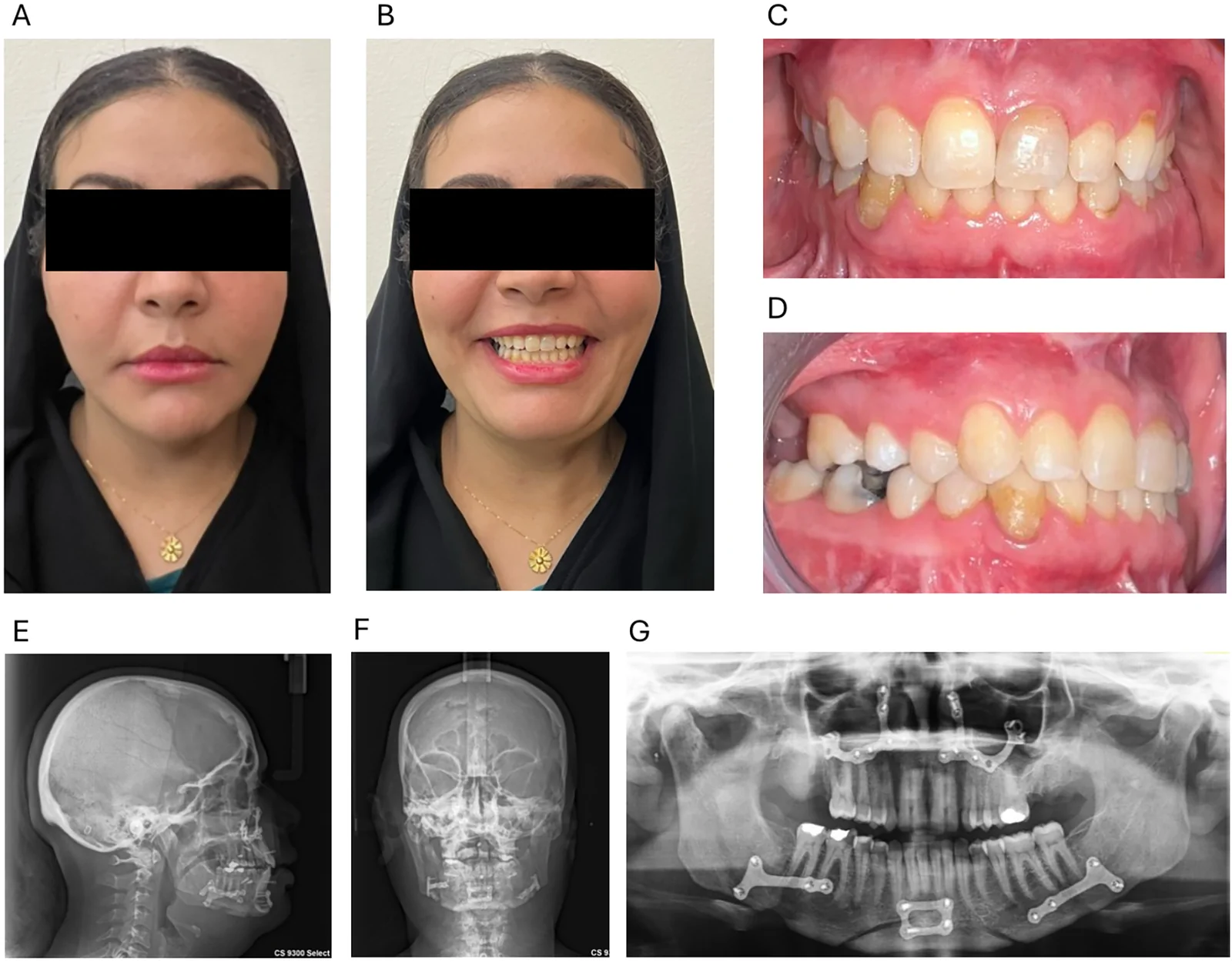
Postoperative extraoral and intraoral photos at the 6-month follow-up visit: (**A**) at rest, (**B**) smile, (**C**) dental midline restored with correction of occlusal canting, (**D**) resolution of the right posterior open bite with ideal intercuspation achieved, and postoperative radiographs obtained on postoperative day 4: (**E**) lateral cephalometric, and (**F**) posteroanterior view. (**G**) Orthopantogram acquired three months postoperatively.

Transitioning from heavy elastics to guiding elastics and removal of preauricular sutures and chin dressing occurred on postoperative day 11 (Figure 4). The maximum interincisal opening progressed from less than a finger width at day 11 postoperatively to two fingers at 2 months and three fingers at 10 and 14 months, with stable occlusal maintenance throughout. At the 6-month follow-up, the occlusion was stable and as set in surgery, with resolution of the open bite and significant improvement in FA (Figures 5A–D). Plain postoperative radiographs were acquired (Figures 5E–G). At the 10-month follow-up, frontal nerve function had returned to normal, and neurosensory testing of the lower lip and chin was consistent with level A. At 14-month follow-up, the patient maintained skeletal symmetry, stable occlusal contacts, and no evidence of relapse or recurrence, and neurosensory testing remained unchanged for the lower lip and chin (level A). No postsurgical orthodontic treatment was done. The patient was subsequently placed on a long-term follow-up regimen with periodic radiographs to monitor for recurrence and ensure a maintained postsurgical stability. Direct patient-reported feedback was not formally obtained. However, based on the surgeon's observations during follow-up visits, the patient appeared satisfied with both functional and aesthetic outcomes.

## Discussion

Osteomas can present as solitary or multiple, slowly growing, bone-forming, sessile or pedunculated overgrowths that are typically asymptomatic (11, 12). The etiology and pathogenesis of osteomas remain unclear (5). While some consider osteomas true neoplasms, others consider them hamartomas (7). Various factors, including inflammation, trauma, chronic infection, neoplasia, metaplasia, surgery, irradiation, endocrine, genetic, and developmental factors, have been proposed as potential etiologies for non-syndromic osteomas (5, 13).

Although most osteomas are known to be sporadic, the rare presence of multiple osteomas should prompt consideration of Gardner's syndrome, an autosomal dominant disease characterized by multiple craniofacial osteomas, intestinal polyposis, epidermoid cysts, lipomas, leiomyomas, and fibromatosis (3, 14). Dental manifestations such as multiple odontomas, supernumerary teeth, and impacted teeth have also been reported in this syndrome (7).

Histologically, osteomas consist of mature, dense, cortical-like bone or, less commonly, cancellous bone (11, 12). Radiographically, they appear as well-circumscribed radiopaque lesions, typically with radiodensity similar to that of adjacent bone (15). When symptomatic, clinical presentation varies depending on size and location. For instance, osteomas in the frontal sinus may displace the eye inferiorly, causing proptosis, whereas those in the ethmoidal sinus may cause lateral displacement (16). Facial deformity or swelling, headaches, sinusitis, nasal discharge, mucocele development, ocular disturbance, and blockage of sinus ostia or infundibula may also be associated with paranasal osteomas (1, 14, 17).

Regarding the location of COs within the jaws, particularly, the mandible, an analysis of 87 cases of mandibular peripheral osteomas reported the following distribution across anatomical subunits: 41.3% in the mandibular body, 21.83% in the condyle, 16.09% in the mandibular angle, 11% in the ascending ramus, 8% in the coronoid process, and 2.2% in the sigmoid notch (2). Thus, the condyle remains a relatively rare site (18). A literature search was conducted using PubMed, Scopus, and Google Scholar. The search strategy included combinations of the following keywords: “condylar osteoma,” “mandibular condyle osteoma,” “peripheral osteoma.” Articles published up to the time of manuscript preparation (October 2025) were considered. Eligible studies included published case reports and case series in English describing osteomas involving the mandibular condyle. Studies were excluded if they involved osteomas in sites other than the mandibular condyle, did not report site, represented other types of osteomas (osteoid osteoma, extra-skeletal osteoma), or lacked clinical or management information. The identified articles were screened for relevance, and data were extracted and qualitatively analyzed to summarize the clinical presentation and management strategies. This literature review revealed 45 reported cases of CO between 1927 and 2025 (Table 1). This structured approach aimed to provide a comprehensive and focused overview of the existing literature on mandibular condyle osteomas. However, it is limited by its narrative design, and no formal systematic review protocol was followed. Patients with CO frequently present with FA, malocclusion, limited mouth opening, trismus, midline shift, mandibular deviation to the unaffected side, and pain (7). These signs and symptoms appear to correlate with the size, progression rate, location, and pressure direction of the tumor (19).

**Table 1:** Cases of CO reported between 1927 and 2025.

Case	Author/year	Age/sex	Type	Location	Trauma	Clinical manifestations	Treatment	Additional procedures
1	Ivy, 1927 (20)	35/F	NR	RC	–	Pain, swelling, MD, malocclusion.	Condylectomy	–
2	Worman et al., 1946 (21)	24/F	C	LC	–	LMO, malocclusion	Condylectomy	–
3	Miles, 1951 (22)	40/F	NR	LC	–	Swelling	NR	–
4	Thoma, 1954 (23)	37/M	C	LC	–	FA, MR, swelling, pain, LMO, malocclusion	Condylectomy	–
5	Nelson et al., 1972 (24)	49/M	P	RC anteromedial surface	+	Swelling	Resection	–
6	MacLennan and Brown, 1974 (25)	31/F	P	L subcondyle	–	Pain, swelling, FA, MD, malocclusion	Subcondylectomy	–
7	Nwoku and Koch, 1974 (26)	34/F	NR	RC	–	FA, malocclusion, LMO	Subcondylectomy	–
8	Wang-Norderud and Ragab, 1976 (27)	35/M	P	LC anteromedial surface	–	FA, mandibular prognathism	Condylectomy	–
9	Weinberg, 1977 (28)	31/M	NR	LC	–	Malocclusion	Condylectomy	–
10	Seymour, 1981 (29)	70/M	NR	RC	–	Stiffness on eating, FA, MD, restricted mandibular movements	Resection	–
11	Papavasiliou et al., 1983 (30)	74/F	P	LC anterior surface	–	Severe pain, trismus, swelling	Condylectomy	–
12	Kondoh et al., 1998 (31)	40/M	P	LC medial surface	–	Malocclusion, FA, MD, LMO	Resection	–
13	Lew et al., 1999 (32)	16/M	P	LC medial surface	–	LMO, jaw tenderness	Resection	–
14	Sayan et al., 2002 (33)	NR/F	P	LC	–	NR	NR	–
15	Chen et al., 2003 (34)	27/M	P	RC	+	FA, malocclusion	Condylectomy	Rib graft
16	Siar et al., 2004 (35)	32/F	P,C	LC	–	LMO	Condylectomy	–
17	Mancini et al., 2005 (36)	19/F	NR	LC	–	FA, MD, malocclusion (canting, crossbite)	Condylectomy	Costochondral graft
18	Ortakoglu et al., 2005 (37)	22/M	NR	RC	–	Malocclusion, MD	Condylectomy	–
19	Woldenberg et al., 2005 (38)	46/M	P	LC	–	Local sensitivity	NR	–
20	Yonezu et al., 2007 (39)	50/M	P	LC lateral surface	–	Pain, swelling, LMO	Resection	–
21	Cogburn et al., 2008 (40)	55/M	P	LC anterior surface	–	Trismus, malocclusion (open bite)	Resection, recontouring of condyle	–
22	Bjornland et al., 2009 (41)	46/M	P	Lateral to condylar neck	+	Swelling, FA	Resection	–
23	Nah, 2011 (42)	29/F	P	C anterior surface	–	NR	NR	–
24	Nah, 2011 (42)	44/F	P	LC anterior surface	–	Difficulty in chewing	NR	–
25	Nah, 2011 (42)	56/F	P	LC anterior surface	–	Difficulty in chewing	NR	–
26	Almeida and de Oliveira Filho, 2011 (43)	45/M	P	RC	–	Swelling, FA, malocclusion, LMO	Resection	–
27	Chaurasia, 2012 (44)	45/F	P	LC neck	–	Swelling, FA	Monitoring	–
28	Kashid and Kumbhare, 2013 (45)	48/M	P	LC anteromedial surface	–	MD, inability to chew, malocclusion, FA, swelling	Resection	–
29	Misra et al., 2013 (6)	22/M	C	LC	–	LMO, swelling, MD	Condylectomy	–
30	Nojima et al., 2014 (19)	46/F	P	LC	–	FA, periauricular discomfort, pain, deviation, protrusion, malocclusion	Resection	Artificial head (silicon)
31	Rajshekar et al., 2015 (46)	35/M	C	RC	+	LMO, FA, MD	Condylectomy	–
32	Fallahi Motlagh et al., 2015 (47)	78/M	P	LC	–	Difficulty swallowing, swelling (preauricular and palatal)	Resection	–
33	Zafar et al., 2016 (48)	60/M	C	RC	–	Pain on mastication, FA, malocclusion, MD	Condylectomy	–
34	De Souza et al., 2017 (49)	67/F	C	RC	–	FA, LMO, pain, MD	Condylectomy	Custom total TMJ prosthesis
35	Heitz et al., 2018 (50)	49/F	P	RC	–	Swelling, LMO	Condylectomy	Myofascial temporalis muscle strip
36	Xu et al., 2018 (51)	32/F	NR	LC	–	LMO	Condylectomy	Single-stage custom TMJ prosthesis
37	Xu et al., 2018 (51)	43/M	P	LC	–	LMO, malocclusion	Condylectomy	Single-stage custom TMJ prosthesis
38	Valente et al., 2019 (7)	52/F	NR	RC	–	Pain, swelling, LMO, malocclusion, MD	Condylectomy	
39	Ostrofsky et al., 2019 (52)	60/M	P	RC	+	Limited mandibular movement, FA, discomfort	Condylectomy	Stock TMJ prosthesis
40	Demircan et al., 2020 (53)	17/M	P	LC	+	Swelling, FA, MD	Resection	–
41	Nilesh et al., 2020 (18)	65/F	C	RC	–	LMO, swelling	Condylectomy	–
42	Tilaveridis et al., 2022 (13)	45/M	P	RC	–	Swelling	Resection	–
43	Tilaveridis et al., 2022 (13)	48/M	P	RC	–	Difficulty chewing, swelling, FA, malocclusion	Resection	–
44	Tilaveridis et al., 2022 (13)	65/M	P	LC	–	Swelling, FA, malocclusion	Monitoring	–
45	Tilaveridis et al., 2022 (13)	42/F	P	LC	–	Swelling, FA, MD, malocclusion	Monitoring	–

F, female; M, male; L, left; R, right; C, condyle; P, peripheral; C, central; NR, not reported; MD, mandible deviation; FA, facial asymmetry; LMO, limited mouth opening; TMJ, tempromandibular joint.

Facial aesthetics are based on many fundamentals, with symmetry being a key determinant of facial harmony. Studies indicate that asymmetry greater than 3 mm or a cant exceeding 3° is readily perceptible by observers (7, 54). Among 43 craniomaxillofacial osteomas, 25.5% presented with FA (55). Of the 18 cases of peripheral COs, 33.3% had FA (13). In the presented table of 45 CO cases, 46.67% of patients with COs had FA (Table 1). The pattern of asymmetry in CO reflects tumor characteristics, onset, muscular compensation, and musculoskeletal development (20). This directs the treatment goal toward complementary correction and realignment rather than the sole removal of the tumor (56).

### Surgical management of condyle osteomas

Small, asymptomatic osteomas generally require no intervention, but periodic follow-up should be emphasized. Symptomatic osteomas presenting with functional and/or aesthetic compromise can be successfully treated by simple surgical excision (56). Most cases of osteomas have excellent prognoses, with low recurrence rates and no risk of malignant transformation (57, 58). However, postsurgical periodic clinical and radiographic follow-up remains essential to detect recurrence or neoplastic changes (5).

In CO cases, small and asymptomatic lesions require no intervention until they reach a size sufficient to encroach on surrounding structures and elicit signs and symptoms (19). When intervention is deemed necessary, management techniques are customized for each case depending on the need to address collateral complications and their severity (51). This may determine whether the treatment is confined to tumor removal alone or extends to reconstruction of the resected mass to restore the affected TMJ apparatus and/or correct the accompanying sequelae of facial deformity and malocclusion (55). Surgical approaches for CO include simple resection or condylectomy (13). If reconstruction is needed, options include autogenous bone grafting with costochondral or sternoclavicular grafts or alloplasts (55). For instance, a total TMJ prosthesis was successfully used in one case to reconstruct the joint after condylar resection due to CO and associated FA (48). Significant improvements in malocclusion and FA were observed following condylectomy with subsequent reconstruction using a rib graft. Orthodontic therapy may be incorporated postoperatively to enhance occlusion and facial balance (34).

OS plays a central role in realigning the facial skeleton, managing maxillofacial dysmorphia, and airway narrowing or obstruction (59). While CO-associated FA may resolve after tumor resection, long-standing lesions can lead to bony remodeling that may require additional corrective surgery to restore facial harmony. Thereby, OS offers an effective means of restoring skeletal symmetry. However, restoring facial symmetry can be quite challenging because of the accompanying variations in hard and soft tissue asymmetries. As a result, additional procedures, such as bone contouring via inferior border ostectomy, bone augmentation, or mandibular angle reduction, may be necessary. Similarly, soft tissue contouring can be performed through masseter muscle and/or buccal fat pad reduction, subcutaneous liposuction, fat graft injection, and face-lifting procedures (60).

Maniskas et al. (61) demonstrated the use of OS performed concurrently with high condylectomy for the management of condylar hyperplasia and the associated FA. Another study reported the use of OS in a case of condylar and ramus osteoma presenting with FA. However, orthodontic treatment and OS were performed 7 years after osteoma resection and were aimed solely at correcting a pre-existing skeletal Class III. In that case, the FA was corrected solely by tumor removal. The delay in OS was justified by the need to prevent a bad split, the young age of the patient (17 years), and the requirement for presurgical orthodontics (51). Another justification for avoiding simultaneous OS was to allow the condyle to occupy the space previously occupied by the excised osteoma (42). Mancini et al. (36) reported persistent FA 1 year after a CO managed with condylectomy alone, with OS eventually planned to address the residual deformity. Nonetheless, the authors emphasized the drawbacks of separate surgeries, particularly the added financial burden (51). Xia et al. (62) reported persistent mandibular asymmetry following combined condylar resection, Le Fort I osteotomy, contralateral mandibular sagittal split ramus osteotomy, and postsurgical orthodontic treatment in the surgical management of condylar osteochondromas associated with mandibular asymmetry. A second-stage procedure involving mandibular body edge osteotomy was subsequently performed because of the space present between the condylar neck and the glenoid fossa. Ultimately, lower facial symmetry improved over 8–12 months due to continued mandibular rotation toward the ipsilateral side.

**Figure 6:**
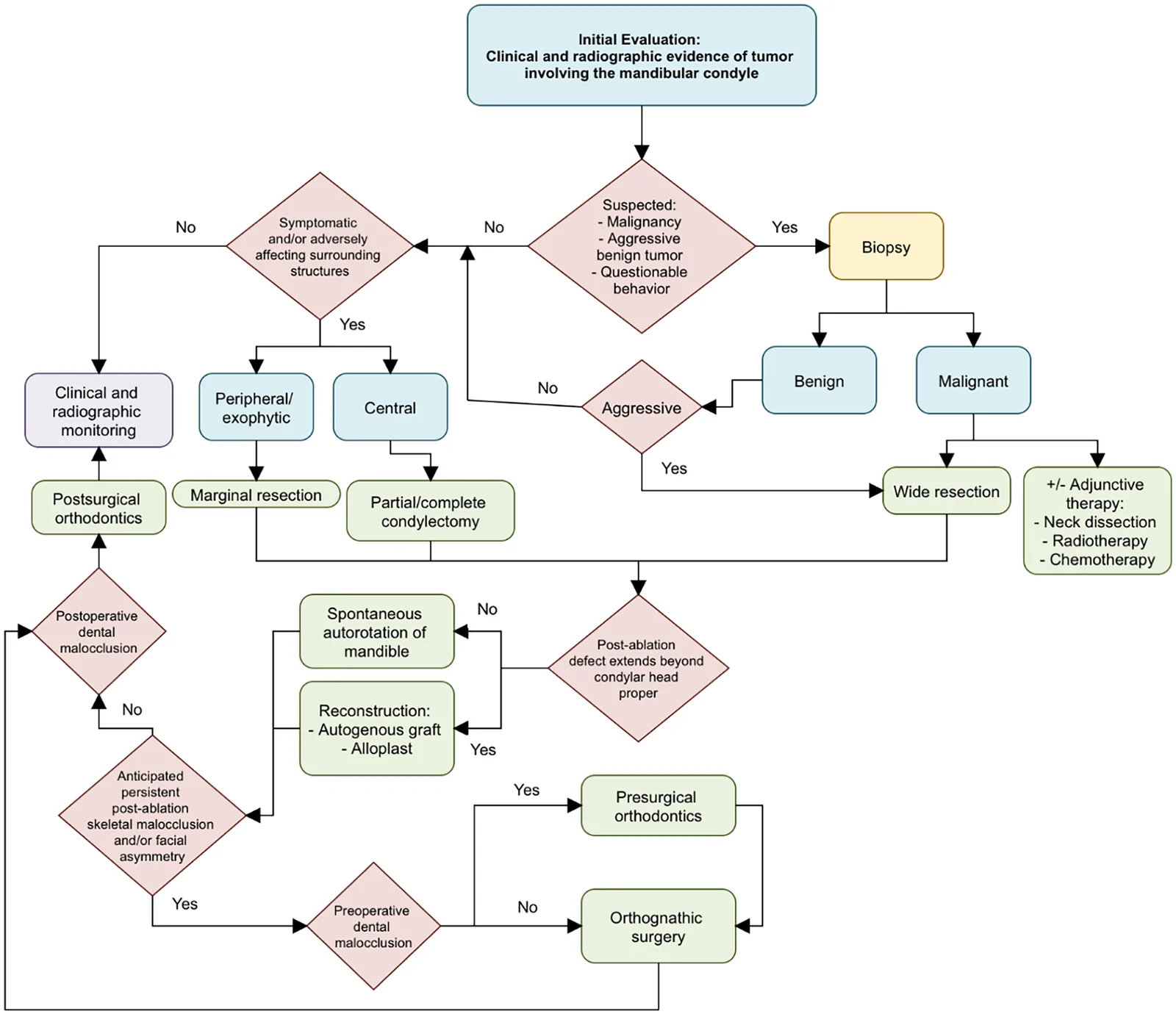
Proposed algorithm for the management of mandibular condyle tumors associated with dentofacial deformities.

A conservative endoscopic approach to the mandibular condyle, performed in conjunction with OS, has been described for the management of condylar osteochondromas using VSP. This minimally invasive intraoral technique avoids manipulation of the facial nerve and parotid gland, thereby minimizing the risks of facial nerve injury, sialocele formation, and visible scarring, while providing enhanced direct visualization within an illuminated surgical field. However, it may be argued that the need to osteotomize the coronoid process during the procedure to facilitate exposure of the condyle suggests that this approach may not be the most conservative option (63). A proposed algorithm for the management of mandibular condyle tumors associated with dentofacial deformities is illustrated in Figure 6.

### Postsurgical stability

Postsurgical changes that reverse outcomes, either through relapse or recurrence of disease, are a major concern following surgery. Given the favorable prognosis of CO, attributed to its benign behavior, characteristic clinical presentation, low recurrence rate following excision, and lack of evidence of malignant transformation, the postoperative course is generally predictable, making a one-stage surgical approach a reasonable option. Skeletal and dental relapse following orthognathic surgery negatively affects postsurgical stability, which is a key measure of surgical effectiveness. A postsurgical dental relapse of ≥2 mm is considered clinically significant, necessitating precise measurement and ongoing evaluation of surgical outcomes (64).

Multiple etiologies have been linked to skeletal relapse and can be broadly classified into presurgical, surgical, and postsurgical factors. Presurgical orthodontics can significantly impact surgical stability, particularly in cases involving complex skeletal discrepancies (64). Kim et al. (65) reported that a surgery-first bilateral sagittal split osteotomy approach was less stable than orthognathic surgery preceded by presurgical orthodontics in patients with skeletal Class III malocclusion. In the present case, the patient demonstrated a skeletal Class I pattern with minor dental malocclusion (open bite, occlusal canting, and dental midline discrepancy). Using VSP, the feasibility of correcting the malocclusion solely through surgery was confirmed, thereby eliminating the need for pre- and postsurgical orthodontics. This decision was further supported by the satisfactory postoperative occlusion achieved. Nonetheless, orthodontic intervention may still be required in cases of CO associated with severe malocclusion or pre-existing complex dentofacial deformities requiring decompensation, alignment, and leveling, all of which may influence postsurgical stability. An example is a condylar osteochondroma case reported by Yu et al. (63) that used presurgical orthodontics for dental alignment and decompensation prior to condylectomy with simultaneous OS. Given the rarity of CO, no studies have explored the outcomes of a one-stage resection combined with orthognathic surgery in such complex cases.

Critical surgical determinants of postsurgical stability include the direction and magnitude of jaw movement, type of procedure, condylar repositioning, type and material of fixation, and surgeon experience. Larger skeletal movements increase biomechanical stress on surrounding tissues, thereby elevating the risk of relapse. Maxillary advancement greater than 7 mm in Le Fort I osteotomy has been associated with significantly higher relapse rates, with horizontal relapse averaging 20.1% and vertical relapse reaching up to 28.4% in some patients. In skeletal open bite cases, isolated mandibular upward repositioning without adequate posterior repositioning may result in relapse of the corrected open bite. Conversely, combined bimaxillary procedures may offer improved functional and skeletal balance. Studies evaluating rigid internal fixation with titanium plates and screws have demonstrated minimal relapse, with a sagittal relapse of 0.65 mm and a vertical relapse of 0.34 mm reported following genioplasty advancement (64). Van Der Wel et al. (66) compared patient-specific osteosynthesis with conventional miniplate fixation in patients undergoing Le Fort I osteotomy. Median translations of less than 1 mm and median rotations of less than 1° were observed, suggesting that both fixation methods provide stable outcomes without significantly affecting postoperative skeletal stability at 1 year of follow-up. In the context of VSP, studies have demonstrated average relapse values of less than 1.8 mm across multiple planes, highlighting its effectiveness in improving surgical precision and enhancing long-term stability (64).

Regarding postsurgical factors, orthodontic treatment, when indicated, plays a crucial role in minimizing the likelihood of relapse. Factors influencing relapse may also be categorized into short-term and long-term contributors. Short-term relapse may result from muscular tension due to high-magnitude surgical movements, condylar dysmorphology, or improper seating of the condyles within the glenoid fossa. Long-term relapse is often associated with gradual skeletal changes, including condylar resorption, continued skeletal growth, and progressive remodeling.

The reporting of long-term outcomes related to CO recurrence and postsurgical stability following combined orthognathic surgery is limited in this study by the relatively short follow-up period of 14 months. However, it is prudent to note that most skeletal relapses develop within the first 12 months postoperatively, as reported in previous studies (67). Therefore, the 14-month follow-up in the present case provides a meaningful timeframe to assess early postsurgical stability and supports a favorable, relapse-free outcome to date, although longer follow-up remains warranted. Additionally, given the benign nature and low recurrence rate of CO, the minimal dental malocclusion, the use of VSP, the implementation of bimaxillary surgery, and the absence of large maxillomandibular movements, a stable postoperative outcome and prolonged disease-free period are anticipated. Nevertheless, long-term clinical and radiographic follow-up remains essential in all benign tumors for early detection of recurrence, potential neoplastic changes, and any associated skeletal relapse or occlusal alterations following orthognathic surgery.

### Role of virtual surgical planning

Given the complexity and delicacy of craniomaxillofacial surgical procedures, rigorous planning free of detrimental flaws was deemed essential. The incorporation of VSP in such cases provided more efficient and accurate outcomes with shorter planning time, offering a superior alternative to traditional surgical planning (68, 69). In addition, 3D-printed individualized titanium surgical osteotomy guides and plates represented another novel modality within OS. They served as stable, accurate, and effective tools for precise cutting and fixation, thereby supporting intricate surgical plans (70). The use of VSP in this case enabled precise delineation of CO margins, simulation of tumor resection, and assessment of the anticipated skeletal and occlusal relationships following surgery. These simulations predicted that tumor resection alone would not fully correct the facial asymmetry. Consequently, the surgical team leveraged the capabilities of VSP to manipulate the maxillofacial skeleton, incorporating an orthognathic surgical plan to achieve facial symmetry and optimal occlusal alignment. By integrating these simulations, VSP facilitated the development of a comprehensive single-stage surgical approach that combined functional and esthetic rehabilitation.

Mandibular CO is a rare entity that exerts significant functional and aesthetic adverse effects on the maxillofacial skeleton and dental occlusion, while also impacting the psychosocial well-being of the patient. This case highlights several strengths, including the ability of a comprehensive single-stage surgical plan combining tumor resection with OS, guided by VSP, to provide functional and esthetic rehabilitation with favorable outcomes and high patient satisfaction. However, limitations must be acknowledged. As a single case report, the findings are not yet generalizable, and the 14-month follow-up period limits the assessment of long-term stability and relapse. Future controlled, longitudinal studies are needed to evaluate the reproducibility and long-term efficacy of this digitally guided, single-stage approach, including occlusal stability, facial symmetry, and patient-reported outcomes, to establish evidence-based guidelines for the management of condylar osteomas.

## Data Availability

The original contributions presented in the study are included in the article/Supplementary Material; further inquiries can be directed to the corresponding author/s.
